# Local delivery of arsenic trioxide nanoparticles for hepatocellular carcinoma treatment

**DOI:** 10.1038/s41392-019-0062-9

**Published:** 2019-09-06

**Authors:** Jian Hu, Yi Dong, Li Ding, Yang Dong, Zhihua Wu, Wenping Wang, Ming Shen, Yourong Duan

**Affiliations:** 1 State Key Laboratory of Oncogenes and Related Genes, Shanghai Cancer Institute, Renji Hospital, School of Medicine, Shanghai Jiao Tong University, 200032 Shanghai, China; 2 Department of Ultrasound, Zhongshan Hospital, Fudan University, 200032 Shanghai, China

**Keywords:** Cancer therapy, Cancer, Drug delivery

## Abstract

Hepatocellular carcinoma (HCC) is a malignancy with a poor prognosis. Surgery combined with chemotherapy has been recommended as a curative regimen for HCC. Nevertheless, the anticancer mechanisms of chemicals in hepatocellular carcinoma remain unclear. Pyroptosis is a type of programmed necrosis, and its mechanism in hepatocellular carcinoma is poorly understood. The efficacy and mechanism of arsenic trioxide nanoparticles in the treatment of HCC were explored in this research. Arsenic trioxide alone and arsenic trioxide nanoparticles were conveniently administered to mice intratumorally using a needle. Compared with As_2_O_3_, As_2_O_3_ nanoparticles (As_2_O_3_-NPs) showed better inhibition, promoted greater LDH release, and induced cell morphology indicative of pyroptosis in vitro. Compared with the free drug, As_2_O_3_-NPs increased GSDME-N expression and decreased Dnmt3a, Dnmt3b, and Dnmt1 expression in Huh7 cells. In vivo, As_2_O_3_-NPs induced a significant decrease in the expression of Dnmt3a, Dnmt3b and Dnmt1, but significantly upregulated the expression of GSDME-N (gasdermin E (GSDME) was originally found to be related to deafness; recently, it has been defined as a gasdermin family member associated with pyroptosis). As_2_O_3_-NPs inhibited tumor growth more strongly than As_2_O_3_ or control, a finding likely attributed to the downregulation of PCNA and DNMT-related proteins and the upregulation of GSDME-N.

## Introduction

Hepatocellular carcinoma (HCC) is a malignancy with a poor prognosis, and an considerable medical challenge.^1^ Surgery is an option to remove or even eliminate the tumor as first-line therapy for cancer patients. A patient can subsequently receive systemic chemotherapy to eliminate residual cancer after surgery, and to keep the cancer from returning.

Many chemotherapeutic agents are used in the clinic. Arsenic trioxide has been used alone and in combination as drug therapy for acute promyelocytic leukemia.^2,3^ In addition, there has been constant progress in applying arsenic trioxide treatment for solid tumors, including HCC.^4–7^ As_2_O_3_ can promote the differentiation of surviving cancer cells. This characteristic allows As_2_O_3_ to reduce the malignant behavior and metastatic risk of surviving cancer cells during chemotherapy, achieving better treatment responses with lower rates of metastasis and recurrence than traditional anticancer drugs.^8^ However, it is difficult to achieve effective As_2_O_3_ accumulation inside a solid tumor because of rapid clearance of this drug in blood circulation.^9,10^ A high dose can be used to maintain the therapeutic activity but causes systemic adverse reactions. Many studies have aimed to determine how to maintain a therapeutic As_2_O_3_ concentration within target solid tumor tissue for an extended period of time with few systemic adverse reactions has been the aim of the most studies. Localized chemotherapy may be an alternative treatment modality. Much attention has been focused on localized chemotherapy, such as a direct intratumoral injection.^11,12^ Local drug delivery systems can prolong the retention time of chemotherapeutic agents at the dosing site to obtain more continuous efficacy and reduce adverse reactions in normal organs and tissues.^13–17^

In the current study, we used mPEG-PLGA-PLL triblock copolymers with cationic amine groups and hydrophobic/hydrophilic chains, which are suitable for carrying drugs.^18,19^ Furthermore, the degradation products of these copolymers are nontoxic to cells.^20^ This study aimed to investigate the nano drug delivery system mPEG-PLGA-PLL loaded with arsenic trioxide, which was delivered via intratumoral administration.

As_2_O_3_ exerts antitumor effects through several mechanisms of action, including the induction of G2/M arrest and apoptosis,^4,21^ but pyroptosis has not been studied in this context. Pyroptosis was originally identified as caspase 1-triggered cell death associated with innate immune defense against microbial infection. In addition, cell death can be induced by caspases 11/4/5, which cleave gasdermin D (GSDMD is a member of the gasdermin family. Gasdermin D has been suggested to act as a tumor suppressor and pyroptosis executor) to trigger pyroptosis.^22,23^ The characteristics of pyroptosis include membrane pore formation, cellular bulging, proinflammatory factor release and cytolysis.^24–28^ GSDME/DFNA5 was originally found to be related to deafness; recently, it has been defined as a gasdermin family member associated with pyroptosis.^25^ GSDME is a pyroptosis execution molecule activated by cleaved caspase 3 after treatment with a proapoptotic agent, such as a chemical drug, that has similar function to GSDMD in membrane pore formation.^24,25^ In this study, we evaluated pyroptosis of Huh7 and HepG2 cells induced by arsenic trioxide formulations for the first time.

## Results

### Preparation and characterization of nanoparticles

**Fig. 1:**
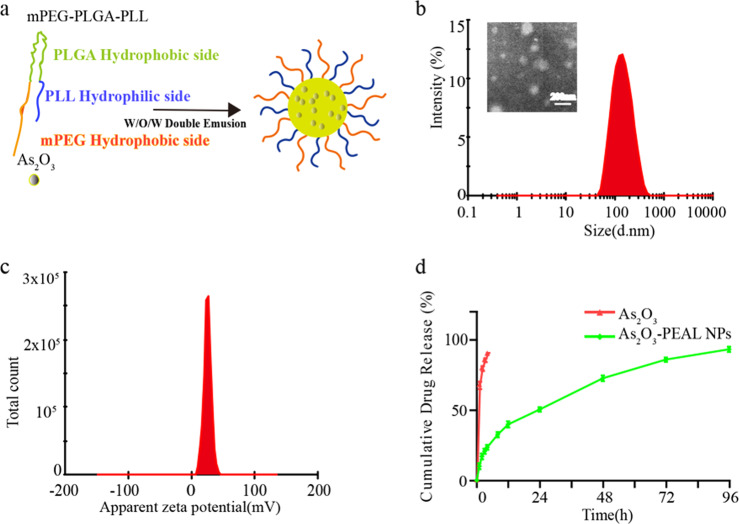
Preparation and characterization of As_2_O_3_-NPs. **a** Schematic illustration of the formulation of As_2_O_3_-NPs. **b** Size distribution and TEM image of As_2_O_3_-NPs. **c** Zeta potential of As_2_O_3_-NPs. **d** Release profile of free As_2_O_3_ and As_2_O_3_-NPs

A double-emulsion method was used to prepare As_2_O_3_-NPs (Fig. 1a). The particle size and PDI (Polymer dispersity index) were 127.5 ± 0.88 and 0.21 ± 0.004, respectively (Fig. 1b). The zeta potential was 26.2 ± 1.42 (Fig. 1c). As shown in the transmission electron microscopy (TEM) image, the As_2_O_3_-PEAL NPs were spherical without aggregation, and the nanoparticle size results were consistent with the DLS results. The loading capacity (DL%) and drug encapsulation efficiency (EE%) of As_2_O_3_-PEAL NPs as measured by ICP were 5.8% and 72%, respectively. The release rate of As_2_O_3_ from As_2_O_3_-NPs was much slower than that of free As_2_O_3_ (Fig. 1d).

### As_2_O_3_-NPs induce HCC pyroptosis

**Fig. 2:**
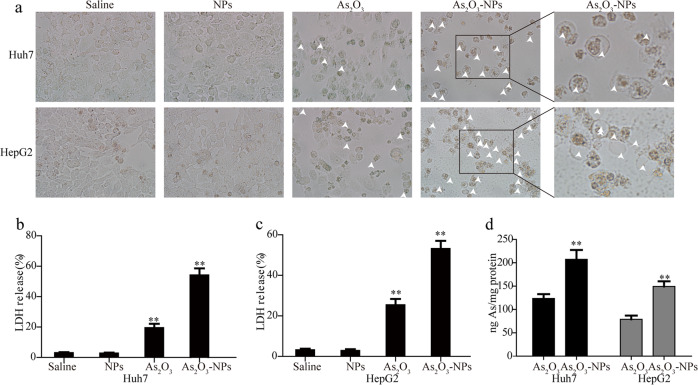
As_2_O_3_-NPs induce HCC pyroptosis. Huh7 and HepG2 cells were treated for 48 h with 4 µM (Huh7) or 1.5 µM (HepG2) As_2_O_3_. **a** Representative microscopic images of Huh7 and HepG2 cells treated with As_2_O_3_ at the indicated concentrations. White arrowheads indicate dying cells with cell membrane blebbing (400×). **b, c** LDH release assays were used to measure cell membrane destruction. **d** Total arsenic uptake was measured by ICP-MS using digested cell pellets. The intracellular arsenic content was normalized to the protein level. The intracellular arsenic content is presented as arsenic (As)/protein (ng/mg). *p < 0.05, **p < 0.01 compared with control

The anticancer effects of As_2_O_3_ were evaluated in the HCC cell lines Huh7 and Bel-7402 (Figs. S2 and S4). Consistently, Huh7 and Bel-7402 cell death was triggered by As_2_O_3_ in a dose-dependent manner. Lactate dehydrogenase (LDH) release was significantly increased in both cell lines with the increase in As_2_O_3_ dose. More dying cells with blebs were observed by phase-contrast microscopy as the dose increased. The As_2_O_3_-NP preparation method was similar to previously reported methods. As_2_O_3_-NP treatment triggered more blebbing cells than free As_2_O_3_ (Fig. 2a). Notably, the LDH release was significantly increased in Huh7 and HepG2 cells treated with As_2_O_3_-NPs compared with free As_2_O_3_ (Fig. 2b, c). The LDH release indicated that As_2_O_3_ destroyed the cell membrane integrity of the HCC cells. The above results showed that As_2_O_3_ treatment caused pyroptosis of HCC cells. The effective uptake of As_2_O_3_ into cells was probably responsible for the high cytotoxicity of As_2_O_3_-NPs. To confirm this hypothesis, we incubated Huh7 and HepG2 cells with free As_2_O_3_ and As_2_O_3_-NPs for 24 h, and the cellular arsenic (As) uptake level was detected by ICP-MS. The cellular uptake level in the As_2_O_3_-NP group was nearly twofold higher than that in the As_2_O_3_ group.

### Therapeutic mechanisms of As_2_O_3_-NPs in vitro

GSDME or GSDMD cleavage can free the N-terminal domain, which inserts into the membrane and triggers pyroptosis. The results indicated that GSDME cleavage was strongly triggered by the increase in As_2_O_3_ dose in Huh7 cells, while GSDMD was not detected by western blotting (Fig. S3). However, in Bel-7402 cells, GSDMD cleavage was induced by increasing doses of As_2_O_3_, and GSDME was not detected (Fig. S5).

**Fig. 3:**
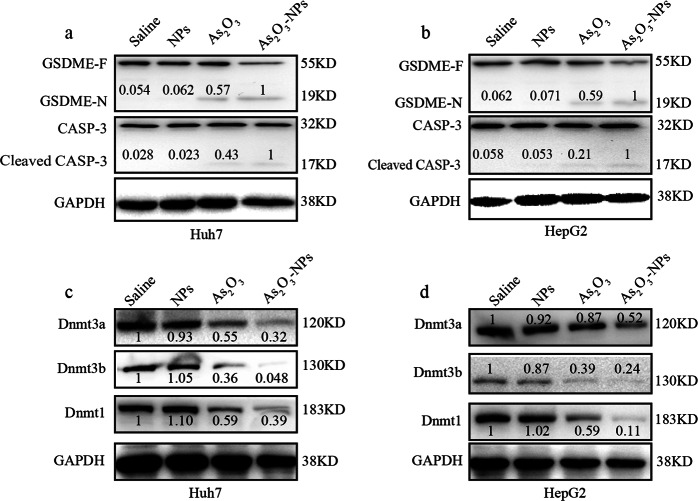
As_2_O_3_ can induce GSDME cleavage. Huh7 and HepG2 cells were treated for 48 h with 4 µM and 1.5 µM As_2_O_3_, respectively. **a** GSDME-F (full-length GSDME), GSDME-N (N-terminal-cleaved GSDME), cleaved caspase 3, and caspase 3 levels in Huh7 cells were evaluated by immunoblotting. (**b**) GSDME-F, GSDME-N, cleaved caspase 3 and caspase 3 levels in HepG2 cells were detected by immunoblotting. **c** Dnmt3a, Dnmt3b and Dnmt1 levels in Huh7 cells were measured by immunoblotting. **d** Dnmt3a, Dnmt3b and Dnmt1 levels in HepG2 cells were detected by immunoblotting

Next, we explored the therapeutic mechanisms of nanoparticles packed with As_2_O_3_ in inducing pyroptosis. GSDME has a gasdermin-N domain capable of inducing pyroptosis in GSDME-expressing cancer cells. GSDME is a key determinant of arsenic trioxide-induced pyroptosis in Huh7 and HepG2 cells. Full-length GSDME (GSDME-F) and N-terminal-cleaved GSDME (GSDME-N) protein levels were detected using western blotting. The results indicated that although both As_2_O_3_-NPs and free As_2_O_3_ increased GSDME-N protein levels, the effect of As_2_O_3_-NPs was more pronounced (Fig. 3). Caspase 3 cleavage was responsible for the As_2_O_3_-induced GSDME activation and pyroptosis. Cleaved caspase 3 levels were increased by As_2_O_3_-NPs compared with free As_2_O_3_.

Studies have shown that arsenic can induce DNA hypomethylation by consuming S-adenosylmethionine, the major cofactor for various methyltransferases, including DNMT, which is responsible for DNA methylation in cells. DNMT-related proteins are associated with patient prognosis and may play an important role in inducing the re-expression of certain proteins. GSDMD expression was induced by As_2_O_3_ in the GSDMD- and GSDME-negative cell line Bel-7402, while GSDME expression was not (Fig. S4). In addition, the expression of Dnmt3a, Dnmt3b, and Dnmt1 was decreased with an increasing dose of As_2_O_3_, which may be responsible for GSDMD expression in Bel-7402. However, GSDMD expression was not induced in GSDME-positive cell lines (Huh7 and HepG2). Immunoblotting of Dnmt3a, Dnmt3b, and Dnmt1 was performed in Huh7 and HepG2 cells treated with the As_2_O_3_ formulation for 48 h. The results showed that As_2_O_3_-NPs and free As_2_O_3_ could reduce the expression of Dnmt3a, Dnmt3b, and Dnmt1, and the effects of As_2_O_3_-NPs were more pronounced (Fig. 3).

### Antitumor effect in the Huh7 xenograft nude mouse model

**Fig. 4:**
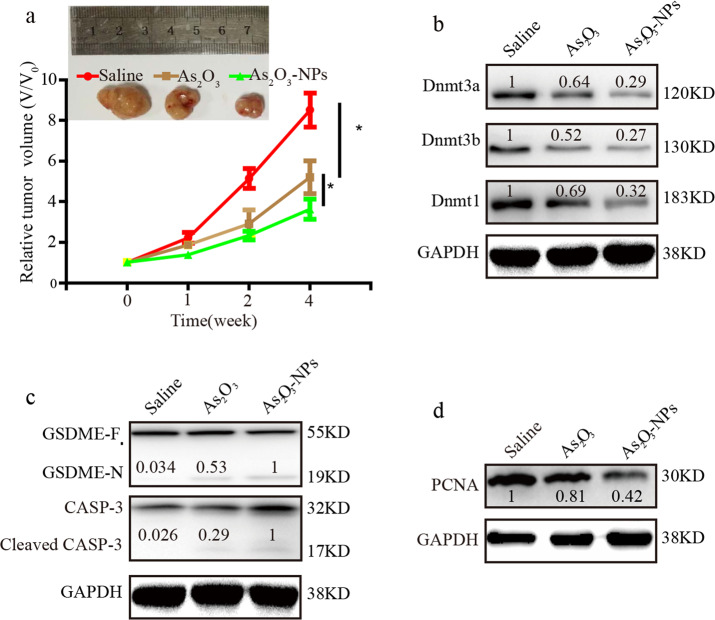
Anticancer effect in the Huh7 xenograft nude mouse model. **a** Change in the relative tumor volume of Huh7 xenograft tumors in nude mouse during the treatment period. **b** The expression of full-length GSDME (GSDME-F), N-terminal-cleaved GSDME (GSDME-N), caspase 3 and cleaved caspase 3. **c** The expression of Dnmt3a, Dnmt3b, and Dnmt1. **d** The expression of PCNA

The in vivo antitumor effect of various As_2_O_3_ formulations on a mouse tumor model established using Huh7 cells was explored. Huh7 xenograft-bearing mice were treated with As_2_O_3_ or As_2_O_3_-NPs through intratumoral administration once a week. Free As_2_O_3_ moderately inhibited tumor growth, while As_2_O_3_-NPs considerably reduced tumor growth (Fig. 4a). Next, the in vivo antitumor activity of different formulations was determined by western blotting. Tumor tissues were homogenized, and proteins were isolated to detect the tumor proliferation marker PCNA by western blotting (Fig. 4d). The protein expression level of PCNA was lower in the As_2_O_3_-NP group than in the As_2_O_3_ or saline group. The increased therapeutic efficacy of As_2_O_3_-NPs in vivo may be due to controlled drug release and the prolonged retention time of As_2_O_3_. Taken together, these experimental data clearly indicated the greatly increased therapeutic effect of the nano delivery system based on its in vivo antiproliferative activity.

The protein expression levels of the pyroptosis-related protein GSDME and DNA methyltransferases (Dnmt1, Dnmt3a, and Dnmt3b) in tumor tissues were evaluated by western blotting (Fig. 4) to explore the mechanism of the in vivo antitumor activity of As_2_O_3_-NPs. GSDME-N expression levels were higher in the As_2_O_3_-NPs group than in the As_2_O_3_ group, which corresponded with the effects of As_2_O_3_-NPs in vitro (Fig. 4c). DNA methyltransferase-related proteins are associated with hepatocarcinogenesis. The protein expression levels of Dnmt1, Dnmt3a, and Dnmt3b were relatively low in the As_2_O_3_-NP group than in the As_2_O_3_ group, a finding that was consistent with the in vitro data (Fig. 4b). The above results showed that As_2_O_3_-NPs may have enhanced antitumor effects through GSDME cleavage and downregulation of DNA methyltransferase expression.

### Safety evaluation of As_2_O_3_-NPs in vivo

**Fig. 5:**
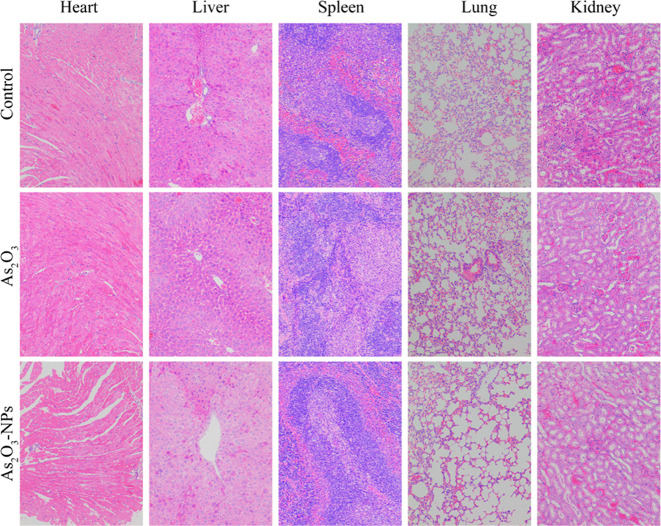
Safety evaluation of As_2_O_3_-NPs in vivo. H&E histology images of heart, liver, spleen, lung, and kidney from tumor-bearing mice after different treatments for four weeks (100×)

Considering the side effects of As_2_O_3_, we evaluated the potential effects of As_2_O_3_-NPs on major organs. Histopathological changes in the heart, liver, spleen, kidney, and lung were analyzed. No significant morphological changes were observed in these organs (Fig. 5). The above results showed that treatment with As_2_O_3_-NPs not only achieved a significant antitumor effect but also had no obvious adverse effects.

## Discussion

Upon systemic injection of an anticancer drug, a small fraction of the drug will reach the solid tumor, and an even smaller amount will penetrate internal tumor tissue and reach the target cells.^29,30^ Local administration allows for the precise delivery of chemical compounds directly to tumor sites.^14^ In this study, we used a PEAL polymer synthesized in our laboratory. Our previous research showed that drug uptake was enhanced by mPEG-PLGA-PLL NPs.^19^ The appropriate size of ~120 nm and the positive charge of the NPs were responsible for their prompt endocytosis. Thus, mPEG-PLGA-PLL packed with As_2_O_3_ exhibited good therapeutic efficacy.

**Fig. 6:**
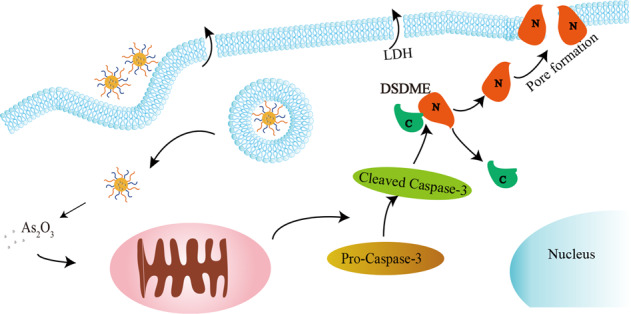
Mechanisms of pyroptosis induced by As_2_O_3_-NPs in HCC. GSDME cleavage is triggered by the caspase 3 activation after As_2_O_3_-NP treatment. The N-terminal domain of GSDME is released and enters the membrane. Next, GSDME-N functions in membrane pore formation, thereby resulting in pyroptosis of As_2_O_3_-treated cells

Arsenic trioxide can downregulate the expression of DNA methyltransferases in cells of various tumors, including HCC. The epigenetic mechanism that has been most intensively studied is the methylation of CpG dinucleotides, which plays a key role in oncogenic transformation and cell fate determination. Most CpG islands in promoter regions in human cancer are hypermethylated, DNA in general is hypomethylated, and transcriptional silencing of tumor suppressor genes occurs.^31–33^ Dnmt3b and Dnmt3a are involved in de novo DNA methylation; however, Dnmt1 plays a key role in maintaining DNA methylation. The hypermethylation of CpG islands has recently been implicated in hepatocarcinogenesis through the epigenetic silencing of tumor suppressor genes. Dnmt1 is the main enzyme responsible for copying methylation patterns after DNA replication.^34^ In HCC patients, elevated Dnmt1 protein expression is related to a significantly lower survival rate compared with decreased Dnmt1 protein expression. Thus, increased Dnmt1 protein expression may play an important role in the malignant progression of HCC, which indicates that it is associated with a poor prognosis in HCC patients.^35^ In this study, As_2_O_3_-NPs also reduced the expression of DNA methyltransferases in vivo.^36,37^ PCNA plays an important role in DNA replication, and As_2_O_3_-NPs decreased PCNA expression. The Dnmt1–PCNA interaction could lead to the prompt remethylation of newly biosynthesized and naked DNA before assembly into chromatin.^38^ Therefore, As_2_O_3_-NPs may show enhanced therapeutic efficacy by decreasing the expression of PCNA and the Dnmt1–PCNA interaction (Fig. 6).

Apoptosis and programmed necrosis have distinct phenotypes. Apoptosis is a universal method of nonlytic cell death, and apoptotic cells have an immunologically ‘silent’ phenotype. Necrotic cells swell and release inflammatory factors, such as IL-1β, leading to lytic cell death.^37^ Pyroptosis is a type of programmed necrosis that depends on GSDME or GSDMD cleavage and is characterized by membrane pore formation, cell bulging, bleb formation, cytolysis, and the release of cytoplasmic content.^25^ The results of our study indicated that As_2_O_3_ induced typical pyroptosis, characterized by large membrane blebs and LDH release from HCC cells. Moreover, the LDH release assay indicated membrane disruption in As_2_O_3_-treated HCC cells. GSDMD has been demonstrated to promote membrane pore formation and induce pyroptosis. As_2_O_3_ induced GSDMD expression and pyroptosis in Bel-7402 cells, perhaps by influencing the methylation of CpG islands on GSDMD through decreased Dnmt1, Dnmt3a, and Dnmt3b expression.

Recently, GSDME was shown to have the same function as GSDMD in pyroptosis. The GSDME gene was originally found to result in autosomal dominant hearing loss (HL). GSDME-related HL is due to a highly specific gain-of-function mutation that increases the activity of GSDME, and the expression of this specific mutant protein causes pyroptosis. Apart from its involvement in HL, GSDME functions as a tumor suppressor. Epigenetic mechanisms result in inactivation of the GSDME gene in many types of cancer, including colorectal, breast, and gastric cancer. While little is known concerning its role in HCC, HepG2 cells express detectable GSDME protein, resulting in the formation of GSDME-N after treatment with cytochrome c. Our study indicated that GSDME expression levels were relatively high in Huh7 and HepG2 cells. As_2_O_3_ induced the cleavage of GSDME. The expression levels of GSDME-N were significantly higher after As_2_O_3_-NP treatment than after As_2_O_3_ treatment alone. GSDME activation in chemical-induced pyroptosis occurs downstream of caspase 3, and GSDME cleavage depends on caspase 3 activation to induce pyroptosis.

In summary, we report for the first time that As_2_O_3_ treatment can trigger pyroptosis in GSDME-expressing HCC cells. Furthermore, our study showed that activated caspase 3 cleaved GSDME to release the free N-terminal domain, which then triggered pyroptosis (Fig. 6). These data provide new insights into the action of As_2_O_3_ in HCC cells, which could be exploited for therapeutic purposes.

## Materials and methods

### Materials

mPEG-PLGA-PLL (PEAL) was prepared as previously described.^19^ Arsenic trioxide was kindly provided by Harbin YIDA Pharmaceutical Co., Ltd (Heilongjiang, China). Protein determination kits and RIPA lysis buffer were obtained from Beyotime Institute of Biotechnology. Antibodies against Dnmt1, Dnmt3a, Dnmt3, and GSDME were purchased from Abcam (Shanghai, China), and GSDMD monoclonal antibodies were supplied by Sigma (USA). Caspase 3, GAPDH, and PCNA monoclonal antibodies were supplied by Cell Signaling Technology (Shanghai, China).

### Preparation and characterization of As_2_O_3_-PEAL nanoparticles

mPEG-PLGA-b-PLL NPs were prepared as previously described. A double-emulsion method was used to prepare As_2_O_3_-PEAL NPs (As_2_O_3_-mPEG-PLGA-b-PLL nanoparticles) as follows. Briefly, a 0.2-ml aqueous solution of As_2_O_3_ (5 mg/ml) was added into 0.5 ml of dichloromethane containing 10 mg of PEAL copolymer and emulsified with an ultrasonic processor at 400 W for 2 min. Next, the above emulsion was added to 1.8 ml of pluronic F68 water solution (1 mg/ml) and emulsified at 400 W for 2 min. Then, the resulting mixture was stirred at room temperature to remove the organic phase. The size distribution and zeta potential of the NPs were determined with a Zetasizer IV analyzer (Malvern Zetasizer Nano ZS90, UK). Morphological evaluation of As_2_O_3_-PEAL NPs was conducted by TEM. The loading capacity (DL%) and encapsulation efficiency (EE%) of As_2_O_3_ in As_2_O_3_-PEAL NPs were measured by inductively coupled plasma emission spectroscopy (ICP) combined with ultracentrifugation (35 min, 18,000 r/min). Unencapsulated As_2_O_3_ in the supernatant was quantified using the ICP method. In vitro As_2_O_3_ release was assayed according to previously published procedures.

### Assessment of pyroptotic cell death under optical microscopy

Huh7 and HepG2 cells were observed for pyroptosis. Huh7 and HepG2 cells were seeded in a 6-well plate (2.25 × 10^5^/well) and cultured for 24 h; then, the medium was removed, and different concentrations of drug (4 µM for Huh7 cells, 1.5 µM for HepG2 cells) were added for 48 h. Representative images were obtained using a microscope (Olympus IX71, Japan).

### The LDH release assay and cellular uptake of arsenic

The LDH release into the medium of cells treated with different formulations, similar to those described above, was determined by an LDH Cytotoxicity Assay Kit (Beyotime). The absorbance was then detected at 450 nm. The release of LDH into the cell culture medium was calculated as a percentage of total LDH release after cell lysis. For the analysis of arsenic influx, two HCC cell lines were treated with the indicated concentration of drug (4 µM for Huh7 cells, 1.5 µM for HepG2 cells) for 24 h, according to previous reports.^39^

### Western blotting analysis of pyroptosis- and DNMT-related proteins in vitro

Total protein was extracted from tumor cells exposed to different treatments. A BCA protein determination kit was used to measure the protein concentration. Ten micrograms of protein from each sample was loaded and separated by SDS-PAGE (10–15%). After transfer to a PVDF (Polyvinylidene Fluoride) membrane, the proteins were analyzed using Dnmt1, Dnmt3a and Dnmt3b, GSDME, GSDMD, caspase 3, and GAPDH antibodies. All monoclonal antibodies were diluted 1:1000. After incubation with the corresponding HRP-conjugated secondary antibodies (1:1000), protein bands were detected. The GAPDH expression level was used as a standard to normalize the expression of the other proteins.

### In vivo antitumor activity of As_2_O_3_-NPs

This study was performed in accordance with the guidelines approved by the Animal Care and Use Committee of the Shanghai Cancer Institute. The xenograft tumor model was established by the subcutaneous inoculation of 2 × 10^6^ Huh7 cells mixed with Matrigel into the backs of nude mice. After the tumor volume reached ~200 mm^3^, the mice were randomized into three groups. The three groups received intratumoral injections of saline, free As_2_O_3_, or As_2_O_3_-NPs (all As_2_O_3_ formulations were equivalent to 2 mg/kg body weight). Each formulation was administered four times at 1-week intervals. The tumor volume was measured before every injection. The tumor dimensions were measured with a caliper to monitor tumor growth. The tumor volume (*V*) was estimated based on the following equation: *V* = [length × (width)^2^]/2.

### Safety evaluation of As_2_O_3_-NPs

Mice in each group were randomly chosen and sacrificed after treatment for 4 weeks. Next, the major organs, including the heart, liver, spleen, lung, and kidney, were removed and fixed in 4% formaldehyde for 48 h. Then, the samples were embedded in paraffin, sectioned, stained with hematoxylin and eosin (H&E) and observed with a digital microscope.

### Statistical analysis

The results are presented as the mean ± SD. The level of significance was determined by one-way ANOVA (analysis of variance) using SPSS software (version 17.0; IBM Inc., Chicago, IL, USA). A *P* value < 0.05 was considered statistically significant, and *P* < 0.01 indicated a highly significant difference.

## Supplementary information

Supplementary Materials

## Data Availability

All data generated or analyzed during this study are included in this published article and its supplementary information files.
