# Supplementary material for: Local delivery of arsenic trioxide nanoparticles for hepatocellular carcinoma treatment
Source: Signal Transduct Target Ther. 2019 Sep 6;4:28. doi: 10.1038/s41392-019-0062-9 (PMC6799825; doi:10.1038/s41392-019-0062-9)
Supplement: Supplementary file 1 — Supplementary Materials [file 41392_2019_62_MOESM1_ESM.docx]

Supplementary Materials for

**Local delivery of arsenic trioxide nanoparticles for hepatocellular carcinoma treatment**

Jian Hu, Yi Dong, Li Ding, Yang Dong, Zhihua Wu, Wenping Wang, Ming Shen*, Yourong Duan*

^*^Correspondence authors. Email: [yrduan@shsci.org](mailto:yrduan@shsci.org) (Y. D); [mshen@shsci.org](mailto:mshen@shsci.org) (M. S)

**This PDF file includes:**

Figures. S1 to S5

**Other Supplementary Materials for this manuscript include the following:**

Meta-data


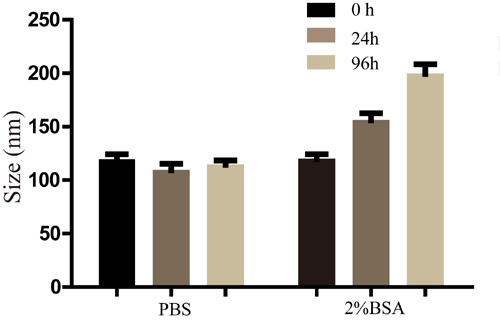


**Figure S1．**Sizes of As_2_O_3_-NPs in PBS and 2% BSA at 37 °C for 0, 24 and 96 h.


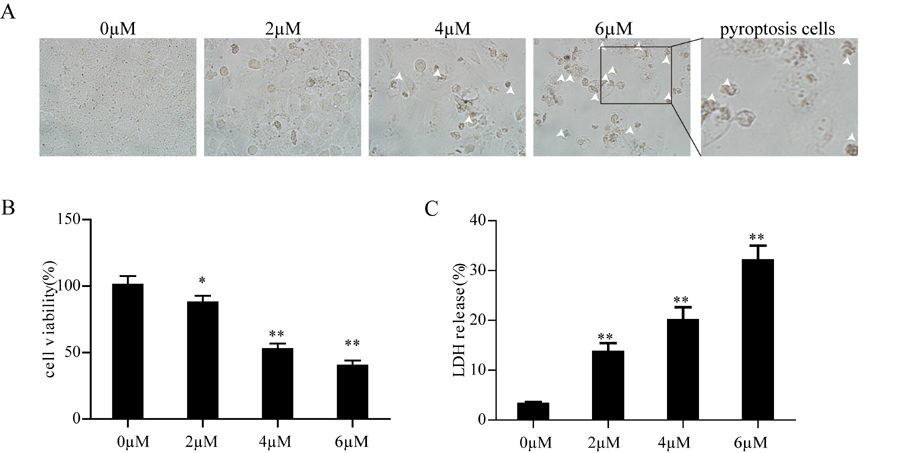


**Figure S2．**As_2_O_3_ induces Huh7 pyroptosis. Huh7 cells were treated with As_2_O_3_ at the indicated concentrations (4 µM) for 48 h. (A) Representative microscopic images of Huh7 cells were treated with As_2_O_3_ at the indicated concentrations. White arrowheads signed the dying cells with balloon-like bubble in the cell membrane (400×). (B) Cell viability of Huh7. (C) Cytotoxicity of Huh7 as measured by lactate dehydrogenase (LDH) release in the culture supernatants. *p < 0.05, **, p < 0.01 compared with the control.


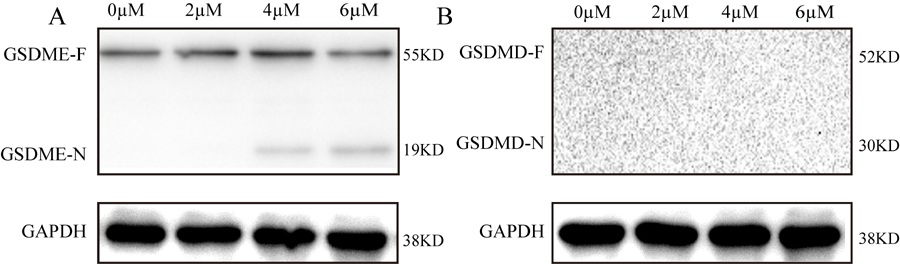


**Figure S3．**As_2_O_3_ can induce GSDME cleavage in Huh7 cells. Huh7 cells were treated with As_2_O_3_ at the indicated concentrations (4 µM) for 48 h. (A) Full-length GSDME (GSDME-F) and GSDME-amino terminal (GSDME-N) were detected by western blotting in Huh7 cells. (B) Full-length GSDMD (GSDMD-F) and GSDMD-N terminal (GSDMD-N) were detected by western blotting in Huh7 cells.


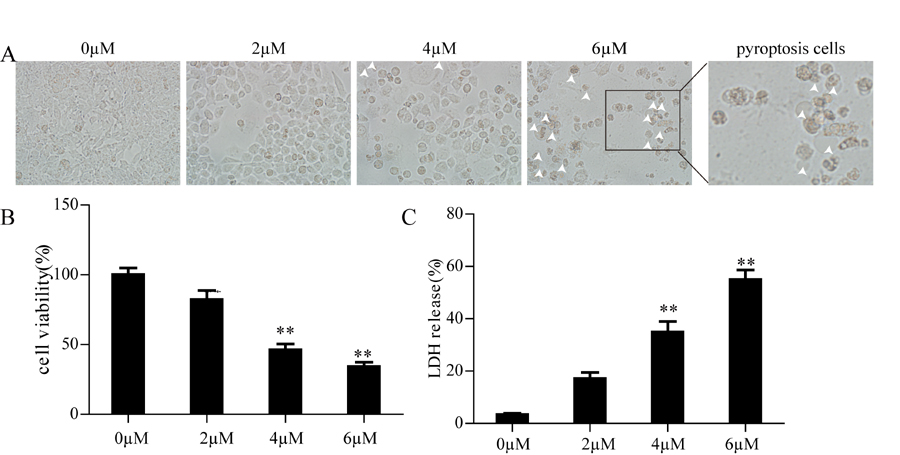


**Figure S4．**As_2_O_3_ induce Bel-7402 pyroptosis. Bel-7402 cells were treated with As_2_O_3_ at the indicated concentrations (4 µM) for 48 h. (A) Representative microscopic images of Bel-7402 cells treated with As_2_O_3_ at the indicated concentrations. White arrowheads signed the dying cells with balloon-like bubble in the cell membrane (400×). (B) Cell viability of Bel-7402 cells. (C) Cytotoxicity of Bel-7402 as measured by lactate dehydrogenase (LDH) release in the culture supernatants. *p < 0.05, **, p < 0.01 compared with the control.


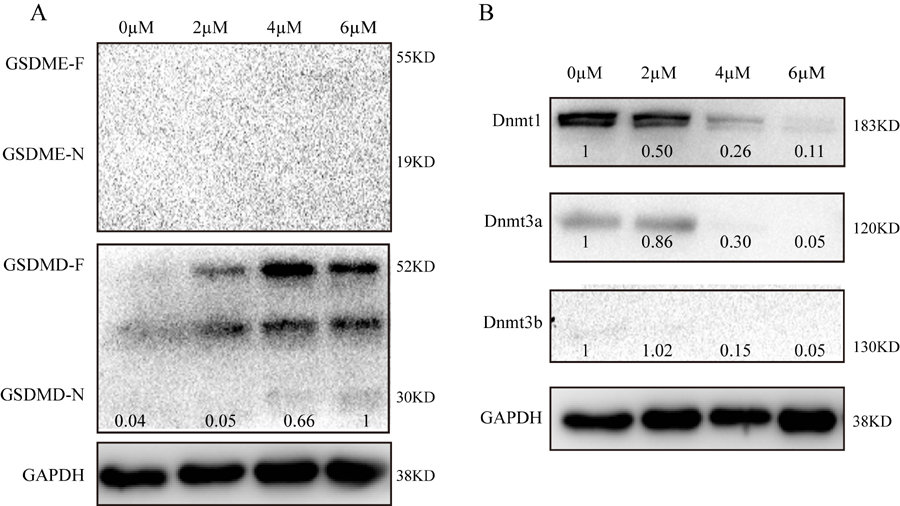


**Figure S5．**As_2_O_3_ can induce GSDME cleavage in Bel-7402 cells. Bel-7402 cells were treated with As_2_O_3_ at the indicated concentrations (4 µM) for 48 h. (A) GSDME and GSDMD were detected by western blotting in Bel-7402 cells. (B) Dnmt1, Dnmt3a and Dnmt3b were detected by western blotting in Bel-7402 cells.
